# Megacystis-Microcolon-Intestinal Hypoperistalsis Syndrome (MMIHS): Series of 4 Cases Caused by Mutation of ACTG2 (Actin Gamma 2, Smooth Muscle) Gene

**DOI:** 10.1155/2021/6612983

**Published:** 2021-03-30

**Authors:** Katarzyna Ignasiak-Budzyńska, Mikołaj Danko, Janusz Książyk

**Affiliations:** Department of Paediatrics, Nutrition and Metabolic Diseases, Children's Memorial Health Institute, Al. Dzieci Polskich 20, Warsaw 04-730, Poland

## Abstract

MMIHS, also known as Berdon's syndrome, is a rare disease that belongs to primary causes of CIPOS (chronic intestinal pseudoobstruction syndrome). Clinical characteristics of MMIHS are differential, but we come across the following classic symptoms: disorders of intestinal peristalsis, microcolon, and megacystis. In this article, we present a series of 4 patients with Berdon's syndrome, in whom we managed to identify the genetic causes of MMIHS. All infants showed clinical features of bowel obstruction and dysfunction of the urinary system after birth. Two of them also manifested disorders from other systems. The prognosis for these patients is poor, but a constant betterment of management in MMIHS, in which the leading role plays TPN (total parental nutrition), causes improvement of patients' survival.

## 1. Introduction

Berdon's syndrome, also known as MMIHS (megacystis-microcolon-intestinal hypoperistalsis syndrome), is a rare motility disorder of gastrointestinal and urinary tract caused by primary dysfunction of the architecture of the smooth muscle cell membrane or intestinal nervous system. The clinical features of MMIHS are various, but we come across three classic symptoms: disorders of intestinal peristalsis, microcolon, and megacystis [1, 2]. The severity of MMIHS and conditions of other organ systems depend on differential variants of genetic mutations that have been identified as molecular causes of Berdon's syndrome. The following genes are known to be involved in pathogenesis of MMIHS: ACTG2 (the most frequent), MYH11 (myosin heavy chain 11), MYLK (myosin light chain kinase), LMOD1 (leiomodin 1), and MYL9 (myosin light chain 9) [3]. However, genetic causes have been identified in approximately 50% of Berdon's syndrome cases. Seven patients with MMIHS have been under treatment at Department of Paediatrics, Nutrition and Metabolic Diseases so far. Here we report a case series of 4 patients with detected ACTG2 gene mutations. The others are without ascertained genetic causes.

## 2. Case 1

**Figure 1:**
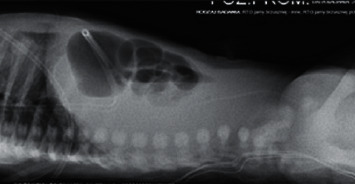
Abdominal X-ray on the first day of life—dilated stomach and bowel loops.

**Figure 2:**
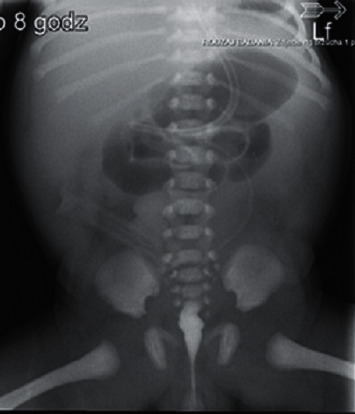
Barium enema revealed microcolon.

A 3-month-old female infant weighing 3050 g with Apgar score of 9 and 8 at 1 min and 5 min was referred to our centre to enter Home Parenteral Nutrition Program (HPN). The girl was born to a non-consanguineous Polish couple and delivered by spontaneous labour at 35 weeks of pregnancy. There were no records of genetic diseases in the family tree. During the ultrasound scan at 22 weeks, megacystis has been noticed in the fetal pelvis with bilateral pelvicalyceal dilatation. At 24 weeks gestation, fetal magnetic resonance imaging (MRI) was performed which revealed a large cystic mass with a maximum diameter of 8 cm and moderate bilateral hydronephrosis. Because of lower urinary tract obstruction, fetal vesico-amniotic shunt (VAS) was implanted at 31 weeks. At birth, there was abdomen distension and absence of intestinal sounds. A few hours after birth, the patient presented food intolerance and gradually developed respiratory distress. The infant has not passed stool for the first 24 hours. Because of these symptoms, intestinal obstruction was suspected. The nasogastric tube for decompression of gastrointestinal tract was inserted, and the central line was implanted to apply total parental nutrition (TPN). Abdominal radiography showed gastric distention and multiple dilated loops of small bowel (Figure 1). Contrast study of upper gastrointestinal tract excluded the mechanical bowel obstruction. Microcolon was detected in the barium enema (Figure 2). The infant underwent exploratory laparotomy on 2nd day of life, in which markedly enlarged intestinal loops extending to 20 cm from Bauhin's valve were discovered. A full-thickness biopsy was taken and ileostomy was performed.

**Figure 3:**
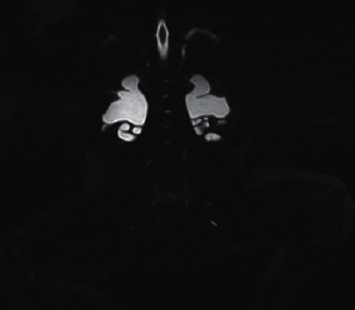
MRI showed bilateral pelvicalyceal dilation and tortuous ureters.

The histological findings were nonspecific (smooth muscle cell vacuolization and insignificant disorders of cytoskeletal architecture). Alpha-smooth muscle actin (alpha-SMA) immunoreactivity was maintained. After surgery, the patient still presented oral feeding intolerance. Stoma has not passed intestinal content, and retention in the nasogastric tube was observed. The rectal biopsy was performed to exclude long-segment Hirschsprung's disease. The histological picture showed normal ganglion cells, and acetylcholinesterase (AChE) activity was normal. Because of persistent motility disorders and oral feeding intolerance, the patient was qualified to decompress gastrostomy tube, and the long-term central venous access (Broviac type) was implanted. Except for gastrointestinal disturbances, the patient was anuric and required protracted catheterization. Ultrasonography (USG) and abdomen MRI showed diminished corticomedullary differentiation, bilateral hydronephrosis, and tortuous ureters (Figure 3). Urodynamics testing revealed hypotonic bladder and detrusor-sphincter dyssynergia. The patient required intermittent catheterization every 3 hours during daytime and night interruption. MMIHS was suspected. The diagnosis was confirmed in genetic tests. Heterozygous de novo missense mutation (c.533G>A/p.Arg178His) was identified in the 5th exon of ACTG2 gene. At present, the girl is one-year-old and has satisfying nutritional status.

She had repeated catheter-related bloodstream infections and one episode of urosepsis.

## 3. Case 2

A 5-month-old female infant with a history of abdominal distension was admitted to our Clinic to continue treatment. The girl was delivered by spontaneous labour at 36 weeks of gestation with a birth weight of 2600 g and Apgar scores of 10 at 1 min and 5 min. This patient was born to a 25-year-old gravida three of a non-consanguineous marriage, with no relevant family history. A second-trimester ultrasound scan showed abdominal cystic structure, moderate bilateral hydronephrosis, and umbilical cord hernia. At 30 weeks of pregnancy, VAS was performed. Initial physical examination disclosed massively distended abdomen and hypoactive bowel sounds. The patient required urinary bladder catheterization. Exploratory laparotomy showed a segment of ileum in which meconium was arrested, but no organic obstruction was found. The umbilical hernia was placed back into the abdominal cavity. Abdominal radiography revealed dilated stomach and nonmajor gas in the distal bowel segments. Because of persistent oral intake intolerance, a central venous catheter was placed into the right internal jugular vein to administer TPN. The infant subsequently developed *Enterococcus faecalis* sepsis and required antibiotic therapy. Because of duodenal obstruction, the patient was operated on 26 days after birth. Ladd's bands and abdominal adhesions were resected, and Santulli stoma was made. Histological findings of the intestinal biopsy revealed vacuolar degeneration of smooth muscle cells. Pathological evaluation of rectal biopsy revealed normal ganglion cells. Percutaneous endoscopic gastrostomy was performed to decompress the stomach. The urodynamics disclosed post-void residual urine, bilateral hydronephrosis, and detrusor-sphincter dyssynergia. The urologists recommended intermittent catheterization and persistent antibiotic prophylaxis.

Genetic testing was performed on the patients and their parents. De novo missense mutation (c.533G>A/p.Arg178His) was identified in the ACTG2 gene in the infant that confirmed MMIHS. At present, the girl is 1.5-year-old, and her growth and development are normal.

She had repeated catheter-related bloodstream infections.

## 4. Case 3

A 9-week-old male infant (2500 g birth weight and with Apgar scores of 6 at 1 min and 5 min after birth) with abdominal distension was referred to our Department to start advanced diagnostic procedures. He was born to healthy non-consanguineous parents and delivered by caesarean section in the 33rd week of pregnancy. The couple did not have the history of hereditary diseases in their family. The boy was delivered by caesarean section in the 33rd week of pregnancy. Prenatal ultrasound studies imaged bilateral obstructive uropathy at 13 weeks, and VAS was implanted at 30 weeks. After birth, the infant presented with a lax abdominal wall, decreased peristalsis, and cryptorchidism. He had poor feeding and bilious vomiting. Conservative treatment (nasogastric tube, intermittent catheterization) and TPN were administered. Abdominal X-ray showed features of small bowel obstruction and a small amount of free gas under the diaphragm.

Because of unresolved intestinal obstruction, surgical intervention (laparotomy) was performed of the 5th day of life. Intraoperative findings revealed vesicointestinal fistula, microcolon, and flaccid bladder. The ileostomy 25 cm far from the ileocaecal valve was performed, and biopsy specimens were taken. Hirschsprung's disease has also been ruled out. The trophic feeding was started to stimulate the sucking and swallowing. The gastrostomy tube for persistent gastric decompression was implanted.

Elevated conjugated bilirubin levels were observed during the first weeks of TPN treatment. After fish oil-based lipid emulsion implementation, the cholestasis was reversed.

Urodynamics testing revealed hypotonic bladder, functional micturition disorders, and vesicoureteral reflux grade 3. Because of this, it was decided to create a vesicostomy.

Patent foramen ovale (PFO) was detected in the echocardiography.

Heterozygous de novo missense mutation (c.188G>A/p.Arg63Gln) was identified in the ACTG2 gene, and the diagnosis of Berdon's syndrome was obtained. At present, the patient is 2.5-year-old and has satisfying nutritional status. He has not developed long-term TPN-associated complications so far.

## 5. Case 4

**Figure 4:**
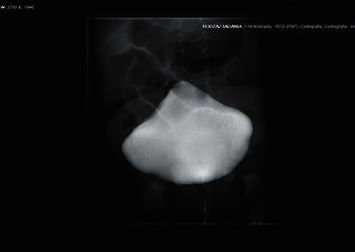
Cystography showed enlarged, hypotonic urinary bladder.

A 6-week-old girl was referred to our Clinic to continue treatment. She was a preterm baby (delivered by caesarean section at 34 weeks of pregnancy with 2500 g birth weighting and with Apgar scores 10 at 1 min and 5 min). The patient was patient was born to a 35-year-old gravida four of a non-consanguineous Polish marriage. The other siblings were healthy, and there were no signs of hereditary diseases in their family tree. During an ultrasound scan at 22 weeks, megacystis with bilateral pyelocalyceal dilation had been noticed. At 29 weeks of pregnancy, VAS was implanted. On initial examination, abdominal distension, abdominal flaccidity, and hyporeactive peristalsis were present. Oral intake induced vomiting. The patient was also anuric. After insertion of the Foley catheter and a nasogastric tube, the urinary bladder and the stomach were decompressed. The obstruction of the gastrointestinal tract was suspected. A central venous catheter was implanted and TPN was started. Upper gastrointestinal tract X-ray examination showed dilated bowel loops and delay in the passage of contrast. The patient was operated on 72 hours after birth. Intestinal malrotation and microcolon with small bowel dilatation up to terminal ileum were seen on laparotomy. The Bishop Koop stoma 15 cm far from the ileocaecal valve was performed, and a full-thickness intestinal biopsy was taken. Voiding cystourethrogram (VCUG) showed a large, distended bladder with bilateral vesicoureteral reflux (Figure 4). The patient has required intermittent catheterization.

Because of the suspicion of deafness, the auditory brain-stem response (ABR) was performed. Conductive hearing loss was ascertained.

Heterozygous de novo missense mutation (c.188G>A/p.Arg63Gln) was identified in the ACTG2 gene, which confirmed MMIHS. At present, the patient is 14-month-old and had recurrent urinary tract infection.

We observed cholestasis with elevated transaminases in all patients during the first weeks of TPN (Table 1). However, after the introduction of trophic feedings and modification of TPN by the implementation of fish oil-based intravenous lipid emulsion, direct bilirubin levels have been normalized. Moreover, the patients (especially patient number 3) have begun to tolerate increased quantities of feeding in the following months, which is not characteristic for patients with Berdon's syndrome.

The prognosis is poor, and all our patients with ACTG2 mutations survive probably to their early adulthood. The life expectancy also depends on occurrence of long-term TPN-associated complications and repeated urosepsis.

## 6. Other Patients with MMIHS

Three of our patients with MMIHS are without ascertained genetic causes. They showed all characteristic symptoms for Berdon's syndrome: disorders of intestinal peristalsis, microcolon, and dilated urinary bladder. However, they have less severe presentation of intestinal dysmotility than patients with detected ACTG2 gene mutations. They tolerated larger portions of enteral feeding, and urinary tract infections occurred sporadically in these patients. However, life expectancy of patients with diagnosed MMIHS is similar, regardless of the gene mutation.

## 7. Discussion

Berdon's syndrome is a rare disease that belongs to the primary causes of CIPOS. According to current literature (in the period 1976–2019), 450 patients with MMIHS were reported. Because of the severity and broad spectrum of possible clinical presentations that accompany cardinal symptoms, management of Berdon's syndrome is complicated. Some of them have been noted in our patients (deafness, blindness, congenital heart defect (PFO), cryptorchidism, and prune belly syndrome). This ambiguousness stems from differential missense or nonsense mutations of genes that code for ingredients of cytoskeletal and play a leading role in smooth muscle contraction [4, 5].

The most frequent disorders are detected in the ACTG2 gene, whose mutations are responsible for 44.1% of cases with known genetic aetiology. The c.533G>A (p.Arg178His) missense alteration of ACTG2 gene, which was in two of our patients, correlates with the most severe presentation of MMIHS (6). The mutation of the following genes: MYH11, MYLK, LMOD1, and MYL9, is also involved in the pathogenesis of Berdon's syndrome in individual cases. However, 50% of patients with MMIHS are without established genetic causes [6–8]. Characteristic prenatal ultrasonography images can suggest MMIHS. The prenatal USG showed megacystis in 88% of the fetus with MMIHS, pelvicalyceal dilation in 66% of prenatally diagnosed cases, or hydramnios [9, 10]. After birth, the newborn presented symptoms of gastrointestinal obstruction and urinary tract dysfunction. Because of this, the initial diagnosis is based on imaging findings, in which microcolon, nonmechanical intestinal obstruction, and dilated urinary bladder are revealed. Despite nonspecific histological changes (vacuolar degeneration of smooth muscle cells, the variability of the number and shape of the ganglion cells in the submucosa and the myenteric plexus, abnormal distribution, or increased connective tissue), the histopathology analysis is also crucial to exclude other possible primary causes of CIPOS.

Berdon's syndrome used to have a fatal prognosis. In the beginning, the mortality rate was approximately 80%, and about 90% of infants died in their first year of life [11]. However, long-term survival rates have increased to 55.6%, respectively (for patients diagnosed with MMIHS in 2004–2011) [12]. It is associated with the improvement of the management of MMIHS and enhanced treatment standards, in which TPN plays a key role. The current evidence confirmed that TPN has a significant impact on prolongation of survival in patients. Unfortunately, individuals develop complications from TPN, including infections, liver failure, thrombosis, etc., which are the main causes of deaths at a later time [13].

The alternative approach is isolated intestinal transplantation or in combination with liver graft. In 1999, Masetti et al. reported the first three patients with MMIHS, who underwent multivisceral transplantation. The patients were alive 17 and 24 months after surgery. One of them died on day 44 of multiple organ failure [14]. The subsequent reports were more promising. In 2013, Huang et al. noticed the case of an eight-year-old boy who was completely off TPN after isolated intestinal transplantation at 4 years of age [15]. Moreover, in 2005, Loinaz et al. described 12 patients with CIPOS (6 with Berdon's syndrome), and 6 of them survived from 2 to 7 years after transplantation. They have begun enteral feeding (after strenuous dietary rehabilitation) and have not required TPN [16]. The analysis of survival after transplantation is hopeful but distant from being satisfying. Because of this, the TPN is currently the most important part of the treatment of MMIHS and would be still a crucial element in the next coming years.

## 8. Conclusions

To sum up, the prognosis of MMIHS is poor, but slow upswing of patients' survival is observed. The management of Berdon's syndrome should be based on improving the quality of nutritional care. At present, the clinician's priority is to avoid long-term TPN-associated complications. Because of unknown molecular causes in most cases, it is a good practice to offer genetic counselling to families with a history of Berdon's syndrome.

## Figures and Tables

**Table 1:** Characteristics of the patients.

Number of patient	The current age (number of months)	The current weight (kg)	The highest level of conjugated bilirubin (mg/dl)	Percentile of weight-for-height	Calories in oral intake (kcal/kg)	Calories in TPN (kcal/kg)
1	11	7.6	1.89	3–10	16.3	88
2	30	11	4.11	3–10	8	86
3	33	15.4	5.56	75–90	84	43
4	9	6.8	11.91	10	15	73

## Data Availability

No data were used to support this study.
